# Tongue stabilisation during CT simulation in radiotherapy using a customised dental biomaterial appliance: a case report

**DOI:** 10.2340/biid.v13.45900

**Published:** 2026-04-23

**Authors:** Khalida Kamarudin, Muhammad Syahir Mohd Suhaimi, Nur Sabrina Zainol Abidin, Wan Adwaa Afiq Wan Zainalam

**Affiliations:** Oral and Maxillofacial Surgery Unit, School of Dental Sciences, Hospital Pakar Universiti Sains Malaysia (HPUSM), Kubang Kerian, Kota Bharu, Kelantan, Malaysia

**Keywords:** Tongue stabilisation, squamous cell carcinoma of the tongue, clear acrylic resin, computed tomography stimulation, radiotherapy

## Abstract

**Introduction:**

Precise tongue stabilisation is essential during radiotherapy (RT) planning to ensure accurate target delineation in patients with squamous cell carcinoma of the tongue. Conventional tongue- depressing techniques can be difficult to apply in completely edentulous patients due to the absence of dentition and limited tongue control. This report aims to present a simple prosthodontic solution for effective tongue stabilisation during RT planning in an edentulous patient.

**Patients:**

A 72-year-old completely edentulous woman diagnosed with stage III (T3N0M0) well-differentiated squamous cell carcinoma of the left side of the tongue was scheduled for definitive RT. Conventional tongue stabilisation methods failed during computed tomography (CT) simulation. A custom-designed clear acrylic resin intraoral appliance was fabricated to maintain a constant 1.5 cm distance between the tongue and the palate and to achieve reproducible tongue depression. Repeated CT simulations were performed to assess the consistency of tongue positioning.

**Results:**

The custom intraoral appliance provided stable and repeatable tongue positioning, allowing improved visualisation of the target volume and facilitating accurate RT planning. Consistent tongue depression was confirmed across repeated CT simulations.

**Conclusion:**

The proposed low-cost, custom-made prosthodontic appliance is an effective method for tongue stabilisation in edentulous patients undergoing head and neck RT. Its use can enhance treatment planning accuracy and may be beneficial in similar clinical situations.

## Introduction

Squamous cell carcinoma (SCC) of the tongue is a common malignancy of the oral cavity, often requiring a multidisciplinary treatment (MDT) approach. Radiotherapy (RT) plays a central role, especially when surgical intervention is not feasible due to the extent of the tumour, functional morbidity, or patient-related considerations. Definitive RT for oral cancer typically involves a total dose of 66–70 Gy, delivered in daily fractions of 2.0 Gy over a period of 6–7 weeks, as recommended by the National Comprehensive Cancer Network (NCCN) and European Society for Medical Oncology (ESMO) guidelines [1, 2]. Accurate computed tomography (CT) simulation is crucial for optimal target delineation and dose distribution. Hence, various tongue depressors or positioning devices are utilised to preserve and replicate the tongue’s position during simulation and treatment sessions. These intraoral devices assist in preventing unintended movement and minimising inter-fraction motion of the tongue while maintaining the position of tumours and adjacent structures during RT delivery. However, maintaining a reproducible tongue position during RT planning and delivery can be challenging in edentulous patients who are unable to retain conventional tongue- depressing devices, which will influence the overall treatment outcomes. This report presents an innovative solution that integrates principles from dentistry and biomaterials to address the challenge and enhance cancer care.

## Patient

**Figure 1:**
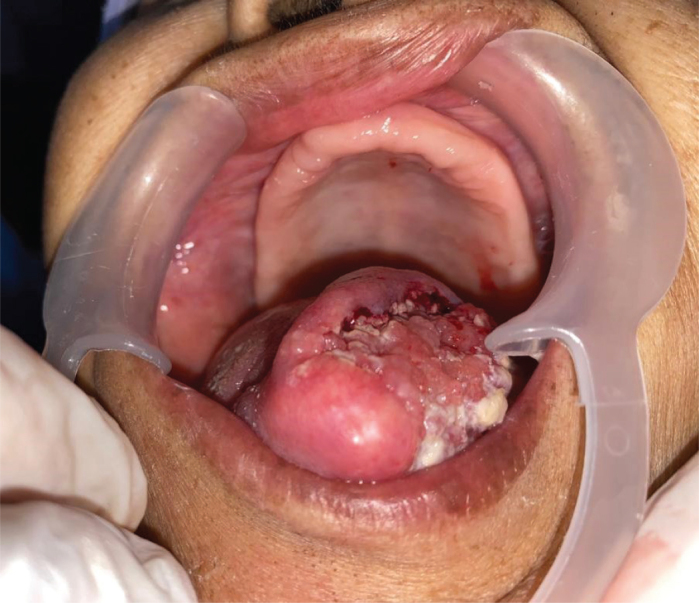
SCC of tongue.

A 72-year-old Malay woman with underlying diabetes mellitus and hypertension was referred to the Oral and Maxillofacial Surgery (OMFS) unit for treatment of a non-healing lesion on her left tongue (Figure 1). A well-differentiated SCC of the left lateral border of the tongue was confirmed by histopathological analysis. Contrast-enhanced computed tomography (CECT) revealed a mass arising from the left side of the tongue measuring approximately 5.0 cm × 2.7 cm × 3.8 cm, with posterior extension to the left tongue base crossing the midline. Subcentimeter left cervical lymph nodes at level II (measuring 7 mm) were noted without radiological features of metastasis. The disease was staged as T3N0M0 (Stage III) according to the American Joint Committee on Cancer (AJCC) 8th Edition of tumour, nodes and metastasis (TNM) staging system for oral cavity carcinoma [3]. In view of the tumour extent and the patient’s advanced age, surgical management would have required total glossectomy with significant functional morbidity. Consequently, definitive RT was planned, and the patient was referred to the Oncology department for further management.

During the initial CT simulation visit, difficulty was encountered in maintaining a still and depressed tongue position. The patient was completely edentulous in both arches and unable to bite or retain a conventional wooden tongue depressor wrapped with wax. The bulky nature of the tumour and poor muscular control associated with edentulism further prevented the formation of an effective oral seal, resulting in the absence of adequate space between the tongue and palate. When planning RT for oral tongue carcinoma, it is crucial to create a repeatable space between the tongue and palate. When the tongue rests against the palate, the interface between the tumour and adjacent structures becomes indistinct on CT imaging, compromising accurate delineation of the gross and clinical target volumes (CTVs). In addition, lack of tongue stabilisation leads to inter- and intra-fraction variability, reducing reproducibility between simulation and daily treatment sessions.

**Figure 2:**
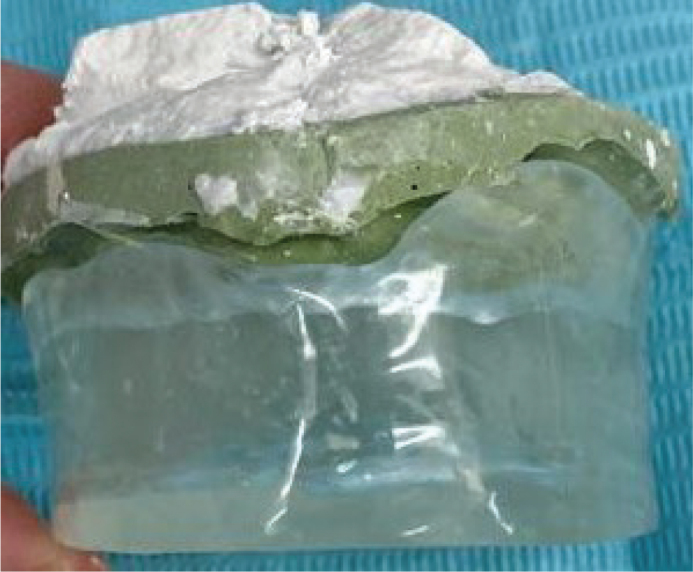
Intraoral appliance (anterior view).

**Figure 3:**
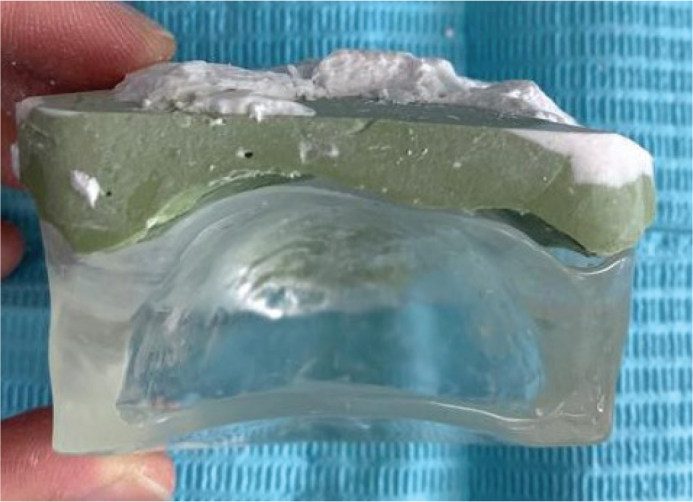
Intraoral appliance (posterior view).

**Figure 4:**
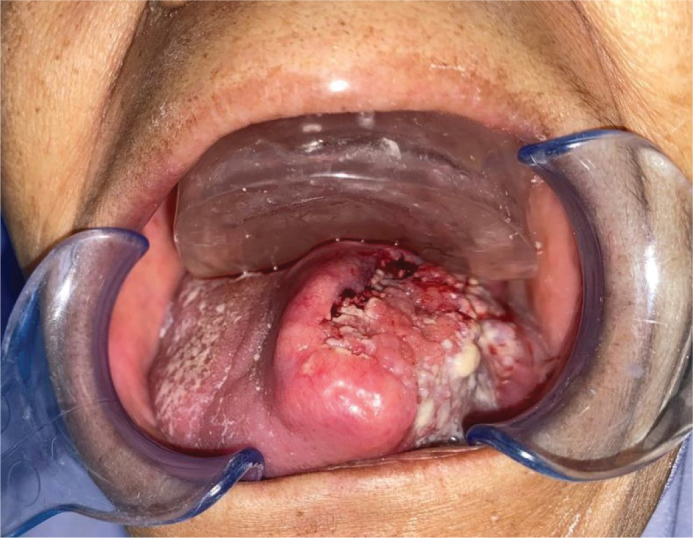
Intraoral appliance issued.

The decision to create a custom intraoral appliance was reached after a multidisciplinary discussion that included the surgeon, oncologist and dental technician. The goal was to maintain a stable tongue position with the tongue and palate separated by about 1.5 cm during the CT simulation and the whole radiation treatment. A clear cold-cure acrylic intraoral appliance was fabricated based on the design (Figures 2 and 3) and issued to the patient (Figure 4). Initially, maxillary and mandibular impressions were taken using alginate. Working casts were created by pouring dental stone into these impressions. Outlines for the palatal base and extension were then drawn on the maxillary cast. Following the application of a separating medium to the cast, a clear cold-cure acrylic resin was adapted onto the palatal surface using the sprinkle-on technique, forming a palatal baseplate. Subsequently, an extended acrylic platform was built, projecting inferiorly from the anterior portion of the baseplate. This acrylic extension served as a tongue depressor, designed to keep the tongue in position during CT simulation. Once the polymerisation process was completed, the appliance was separated from the cast, and any sharp or uneven borders were trimmed and refined using acrylic burrs. Finally, the appliance was disinfected before being trialled and issued to the patient.

## Result

**Figure 5:**
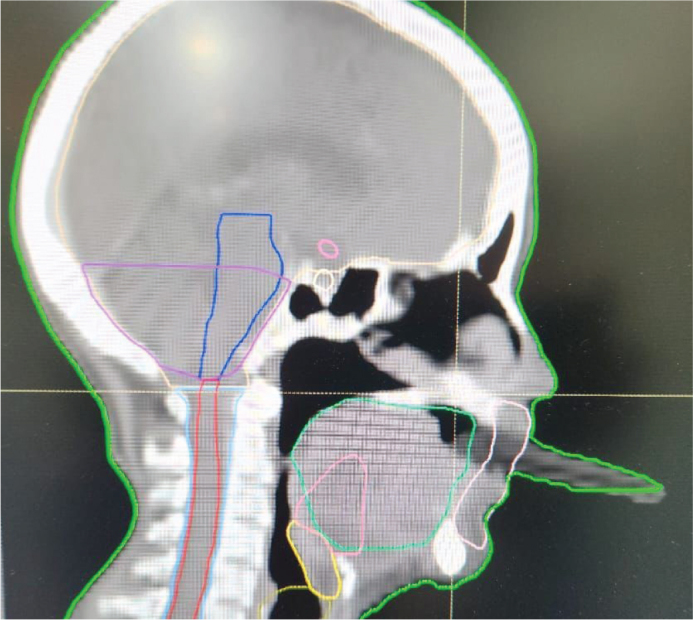
CT simulation tongue depressor.

**Figure 6:**
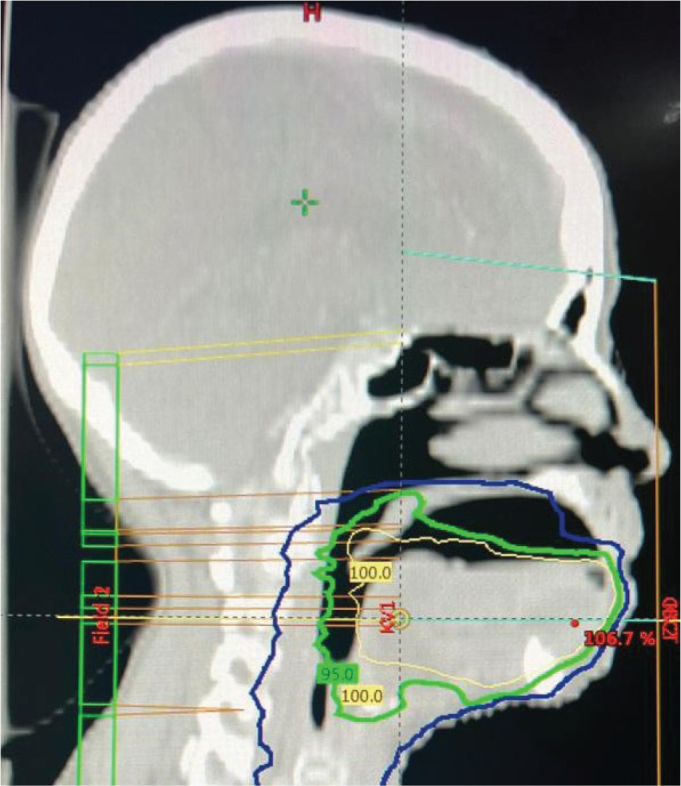
Initial CT simulation with intraoral appliance.

**Figure 7:**
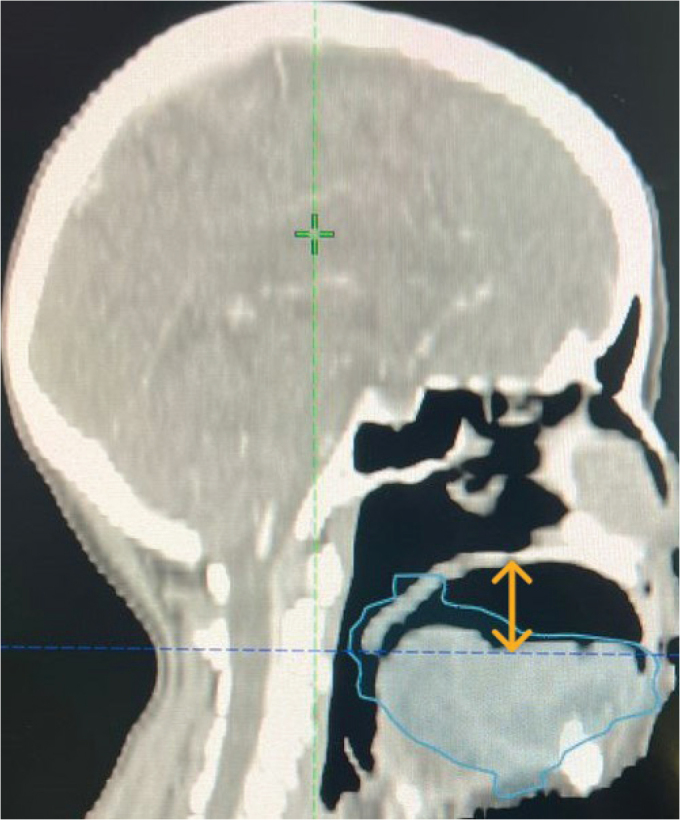
Repeat CT simulation with intraoral appliance.

During CT planning, the platform maintained a consistent 1.5 cm space between the tongue and palate and delicately depressed the tongue upon mouth closure, thereby enabling the clear delineation of oral cavity structures. The patient was provided with comprehensive instructions on the insertion and removal of the appliance, which were similar to the methods used to handle conventional dentures. To prevent trauma to the tongue lesion during placement and removal, additional precautions were implemented. The use of topical lignocaine gel was prescribed in the event of oral mucositis during treatment to enhance patient comfort, and a saliva substitute was advised as a lubricant. Another CT simulation was conducted with the intraoral appliance in place. The repeat scan demonstrated a sufficiently depressed tongue position with an evidently appreciable and reproducible space between the tongue and palate, in contrast to the initial simulation without the appliance. This facilitated the enhanced visualisation and delineation of target volumes and adjacent normal structures. Figures 5–7 present representative CT images demonstrating tongue positioning during simulation: Figure 5 with the wooden tongue depressor, Figure 6 showing the tongue position achieved with the intraoral appliance during the initial CT simulation and Figure 7 showing the reproducible tongue positioning in the subsequent repeat simulation. The customised appliance was well tolerated during CT simulation, and there were no reports of discomfort, gag reflexes or mucosal irritation experienced by the patient.

## Discussion

Modern RT for tongue carcinoma is typically delivered using intensity-modulated radiation therapy (IMRT) or volumetric modulated arc therapy (VMAT) [4]. Both approaches allow highly conformal dose distributions while sparing adjacent critical structures such as the bones, salivary glands and oral mucosa. To ensure optimal oncological control and minimise treatment-related morbidity, the accurate delineation of gross tumour volume (GTV), CTV and organs at risk (OARs) during CT simulation is therefore essential [5, 6]. During CT simulation, patients are immobilised using thermoplastic head-and-neck masks and reproducible intraoral positioning is required throughout the entire course of RT, which commonly consists of daily fractions delivered over several weeks. Variations in tongue position between simulation and treatment sessions may result in suboptimal dose coverage of the target or increased radiation exposure to uninvolved structures. This challenge is amplified in edentulous patients, who lack the support for retention when using existing intraoral tongue-positioning devices such as stock depressors and thermoplastic bite blocks due to the absence of ideal occlusion to bite, which limit the reproducibility of tongue position. Although commercially manufactured stents and 3D-printed patient-specific stents provide improved positional control and enhanced anatomical conformity, a higher cost, extended manufacturing period and the need for specialised digital workflows and tools constraint their broader clinical implementation [7].

The custom acrylic resin appliance described herein provides a patient-specific, radiolucent and dimensionally stable solution that can be produced using standard laboratory methods, resulting in an accessible and cost-effective biomedical device for consistent tongue positioning throughout the treatment process. On top of that, clear cold-cure acrylic resin (polymethyl methacrylate) was selected for its construction due to its biocompatibility, functioning optimally in the warm, moist environment of the oral cavity and reliable engineering properties, reflected by the high modulus elasticity and ability to maintain thickness, geometrical stability and spatial relationships over time, despite exposure to saliva, mechanical stress and humidity. The radiolucent characteristic minimises artefact formation during CT imaging, thereby facilitating accurate delineation of soft tissue structures throughout the fractionated radiation therapy course. The simplicity of manipulation and adaptability enable the fabrication of patient-specific devices without requiring complex manufacturing processes or specialised equipment. Furthermore, the smooth, polishable surface finish and low material weight enhance intraoral tolerance, which is particularly relevant in elderly patients and those susceptible to radiation-induced oral mucositis. These characteristics collectively establish the appliance as a functional biomedical device that enhances standard external immobilisation systems and improves the geometric reproducibility of RT delivery in edentulous patients with tongue carcinoma.

## Conclusion

This case demonstrates that a custom-made intraoral acrylic resin appliance can provide a reproducible, patient-specific solution for tongue immobilisation during RT in completely edentulous patients. The appliance represents a low-cost, accessible and practical adjunct to standard immobilisation methods, particularly in resource-limited settings, and may help improve treatment accuracy and patient outcomes in challenging clinical scenarios.

## Data Availability

Data sharing does not apply to this article, as no datasets were generated or analysed in the current study.
